# Rapid urine-based screening for tuberculosis to reduce AIDS-related mortality in hospitalized patients in Africa (the STAMP trial): study protocol for a randomised controlled trial

**DOI:** 10.1186/s12879-016-1837-z

**Published:** 2016-09-22

**Authors:** Ankur Gupta-Wright, Katherine L. Fielding, Joep J. van Oosterhout, Douglas K. Wilson, Elizabeth L. Corbett, Clare Flach, Krishna P. Reddy, Rochelle P. Walensky, Jurgens A. Peters, Melanie Alufandika-Moyo, Stephen D. Lawn

**Affiliations:** 1 Department of Clinical Research, London School of Hygiene & Tropical Medicine, London, UK; 2 Malawi-Liverpool-Wellcome Trust Clinical Research Program, University of Malawi College of Medicine, Blantyre, Malawi; 3 Department of Infectious Disease Epidemiology, London School of Hygiene & Tropical Medicine, London, UK; 4 University of the Witwatersrand, Johannesburg, South Africa; 5 Dignitas International, Zomba, Malawi; 6 Department of Medicine, College of Medicine, University of Malawi, Blantyre, Malawi; 7 Department of Internal Medicine, Edendale Hospital, University of KwaZulu-Natal, Pietermaritzburg, South Africa; 8 Division of Pulmonary and Critical Care Medicine, Massachusetts General Hospital, Boston, MA USA; 9 Divisions of General Medicine and Infectious Disease, Massachusetts General Hospital, Boston, MA USA; 10 The Medical Practice Evaluation Center, Department of Medicine, Massachusetts General Hospital, Boston, MA USA; 11 Harvard University Center for AIDS Research, Harvard Medical School, Boston, MA USA; 12 The Desmond Tutu HIV Centre, Institute of Infectious Disease and Molecular Medicine, Faculty of Health Sciences, University of Cape Town, Cape Town, South Africa

**Keywords:** TB, HIV, HIV-associated TB, Screening, LAM, Xpert

## Abstract

**Background:**

HIV-associated tuberculosis (TB) co-infection remains an enormous burden to international public health. Post-mortem studies have highlighted the high proportion of HIV-positive adults admitted to hospital with TB. Determine TB-LAM and Xpert MTB/RIF assays can substantially increase diagnostic yield of TB within one day of hospital admission. However, it remains unclear if this approach can impact clinical outcomes. The STAMP trial aims to test the hypothesis that the implementation a urine-based screening strategy for TB can reduce all cause-mortality among HIV-positive patients admitted to hospital when compared to current, sputum-based screening.

**Methods:**

The trial is a pragmatic, individually randomised, multi-country (Malawi and South Africa) clinical trial with two study arms (1:1 recruitment). Unselected HIV-positive patients admitted to medical wards, irrespective of presentation, meeting the inclusion criteria and giving consent will be randomized to screening for TB using either: (i) ‘standard of care’- testing of sputum using the Xpert MTB/RIF assay (Xpert) or (ii) ‘intervention’- testing of sputum using Xpert and testing of urine using (a) Determine TB-LAM lateral-flow assay and (b) Xpert following concentration of urine by centrifugation. Patients will be excluded if they have received TB treatment in the previous 12 months, if they have received isoniazid preventive therapy in the last 6 months, if they are aged <18 years or they live outside the pre-specified geographical area. Results will be provided to the responsible medical team as soon as available to inform decisions regarding TB treatment. Both the study and routine medical team will be masked to study arm allocation. 1300 patients will be enrolled per arm (equal numbers at the two trial sites). The primary endpoint is all-cause mortality at 56 days. An economic analysis will be conducted to project long-term outcomes for shorter-term trial data, including cost-effectiveness.

**Discussion:**

This pragmatic trial assesses an intervention to reduce the high mortality caused by HIV-associated TB, which could feasibly be scaled up in high-burden settings if shown to be efficacious and cost-effective. We discuss the challenges of designing a trial to assess the impact on mortality of laboratory-based TB screening interventions given frequent initiation of empirical treatment and a failure of several previous clinical trials to demonstrate an impact on clinical outcomes. We also elaborate on the practical and ethical issues of ‘testing a test’ in general.

**Trial registration:**

ISRCTN Registry (ISRCTN71603869) prospectively registered 08 May 2015; the South African National Controlled Trials Registry (DOH-27-1015-5185) prospectively registered October 2015.

## Background

HIV-associated TB remains an enormous burden to international public health, even in regions with high coverage of antiretroviral therapy (ART). Globally, in 2014, there were an estimated 0.4 million TB related deaths in people living with HIV, which accounts for approximately one-quarter of TB deaths and one-third of HIV deaths [1]. This burden disproportionately affects sub-Saharan Africa where TB is a common cause of hospital admission and mortality among HIV-positive patients admitted to hospital [2].

Diagnosis of TB in people living with HIV remains challenging due to non-specific clinical features, early dissemination beyond the lungs and relatively low mycobacterial burden within sputum samples [3–5]. A meta-analysis of post-mortem studies in adult HIV-positive patients dying in hospitals in sub-Saharan Africa reported that between 32 and 67 % (pooled summary estimate 43 %) had evidence of TB at post-mortem [6]. TB was disseminated in almost 90 % of patients, and remained undiagnosed at the time of death in almost one-half of TB cases, reflecting a failure of current sputum and clinical based diagnosis of TB, and presenting a strong rationale for routine systematic screening of HIV-positive hospital admissions.

New diagnostic tools have been high on the TB research agenda for the past decade, and are recognised as crucial to the World Health Organization’s (WHO) End TB Strategy [7]. The Xpert MTB/RIF rapid molecular assay (Xpert, Cepheid, Sunnyvale, CA, USA) has a pooled sensitivity for diagnosis of pulmonary TB in HIV-positive adults of 79 % (95 % CI 70–86 %), with 99 % specificity. The test has been approved by WHO and widely implemented in high burden settings [8]. Systematic reviews have also reported very high specificities for Xpert when testing a wide-variety of non-respiratory clinical samples, despite culture being an imperfect reference standard for extra-pulmonary TB [9, 10]. Although data were insufficient for the WHO guidelines to endorse the use of Xpert for TB diagnosis from urine, studies have demonstrated useful diagnostic yield and high specificity in urine among hospitalised HIV-positive patients [11–14].

Urine also has several advantages as a diagnostic sample for hospitalised HIV-positive patients, including relative ease of collection and lower biohazard risk during specimen handling during collection and in the laboratory. The Determine TB-LAM (TB-LAM, Alere, Waltham, MA, USA) lateral flow assay is a simple, point-of-care test for detecting the mycobacterial cell wall antigen lipoarabinomannan (LAM) in urine. It requires 60 μL of unprocessed urine, giving a result in 25 min at a relatively low cost (approximately US$2.50). Whilst sensitivity of this assay is poor in general populations, it is improved in advanced HIV-related immunosuppression, and studies in HIV-positive patients admitted to hospital have demonstrated sensitivities between 40 and 70 % [15–20]. Specificity is exceptionally high when the reference standards include culture of non-respiratory samples [19, 21]. TB-LAM was conditionally approved in November 2015 by WHO for use in diagnosing TB in hospitalised HIV-positive patients [22].

Evidence suggests that high mortality amongst hospitalised patients with HIV/TB co-infection is fuelled by under-diagnosis of TB and delays in diagnosis due to overreliance on sputum based diagnostics, imaging and/or clinical features, and an inability to diagnose disseminated and extra pulmonary TB disease. We therefore sought to evaluate the impact on mortality of a high-yield, rapid-urine based screening approach for TB in HIV-positive medical admissions to hospital in South Africa and Malawi.

### Rationale for studies of clinical impact

A study from South Africa intensively screened unselected HIV-positive hospital admissions to medical wards with comprehensive clinical sampling (sputum, blood, urine and other clinically relevant samples) [14]. Using mycobacterial liquid culture and/or Xpert, TB was diagnosed in 139/427 patients (33 % TB prevalence, 95 % CI 28–37 %). However, only 28 % of microbiologically confirmed TB in this study could have been diagnosed by Xpert testing of sputum alone. In contrast, 81 % of TB cases could have been diagnosed by Xpert testing of both sputum and urine, with additional TB-LAM testing of urine, and results are available within the first 24 h of hospital admission [14, 23]. Xpert is the best diagnostic test available in most high-burden settings and the WHO’s recommended initial TB test for HIV-positive patients. The use of this rapid, relatively low cost screening approach increased diagnostic yield by almost three-fold.

Despite this increase in diagnostic yield, there is no evidence that this urine-based screening strategy will impact mortality or clinical outcomes; it is recommended that impact be demonstrated before such interventions can be endorsed or implemented [24]. This is especially true, as numerous studies have noted that the replacement of sputum smear-microscopy with the more sensitive Xpert has failed to demonstrate any impact on clinical outcomes [25]. A recently published randomised controlled trial of adjunctive urine testing with the TB-LAM assay (the LAM RCT) in a selected population of hospitalised HIV-positive patients being investigated for TB demonstrated an absolute mortality reduction of 4 % (95%CI 1–7 %) and a relative reduction in mortality of 17 % (95%CI 4–27 %). These data support the potential for a urine-based screening approach to reduce mortality among unselected HIV-positive in-patients [26].

### Rationale for a Randomized Controlled Trial (RCT)

Whilst a high yield, urine-based screening strategy might benefit this patient population by increasing the number of TB diagnoses and decreasing time to TB treatment, it may also be associated with a range of adverse consequences. A rapid TB diagnosis within the first 1–2 days of hospital admission may divert clinical attention from co-pathologies, and may reduce the likelihood of empirical prescription of antibiotics for occult bacterial infections. Further, despite excellent specificity, this screening approach may lead to some false-positive TB diagnoses and false-positive rifampicin resistance results from Xpert assays, leading to inappropriate use of potentially harmful medication. Although relatively low cost, the implementation of such a screening strategy will require investment of limited health service resources, and may divert resources from other interventions with potentially greater impact on health outcomes.

Although diagnostic tests are routinely implemented on the basis of diagnostic accuracy or effectiveness without assessment of impact on clinical outcomes (e.g. Xpert and rapid malaria diagnostics), their impact on population health is part of a wider cascade of processes including care seeking and initiation of appropriate treatment [27]. Thus, they should be evaluated under such circumstances too, and evidence of diagnostic accuracy alone should not be taken as evidence of impact on patient-appropriate outcomes such as reducing morbidity or mortality [27]. This is especially true of TB diagnostics, as even mycobacterial culture (the most sensitive diagnostic) can have minimal impact on clinical decision to initiate TB treatment [28].

## Methods/Design

### Aim

The principal aim of this trial is to test the hypothesis that the implementation of a rapid, sensitive urine-based screening strategy for TB can reduce all cause-mortality among HIV-positive patients admitted to hospitals in sub-Saharan Africa when compared to current, sputum-based screening.

### Study design

**Fig. 1:**
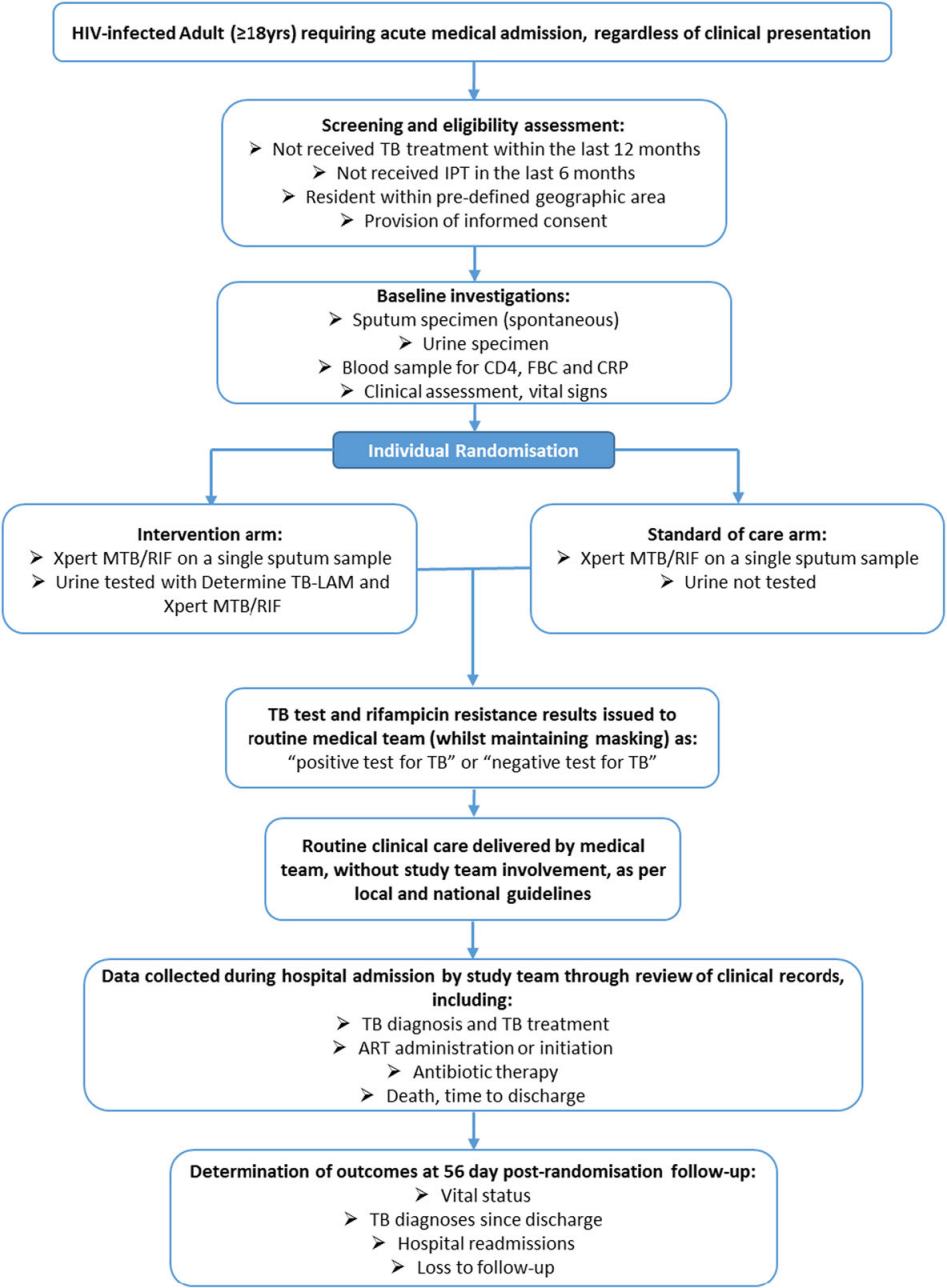
Study design flow diagram

The trial is a pragmatic, individually randomised, multi-country clinical trial with two study arms with 1:1 allocation between arms (Fig. 1). HIV-positive patients randomised to the ‘standard of care’ arm will be screened for TB by testing sputum (if produced by spontaneous expectoration) using the Xpert assay. HIV-positive patients randomised to the intervention arm will, in addition to the standard of care, have a urine sample (if produced) tested for TB using the TB-LAM assay and, following concentration of 40–50mls of urine by centrifugation, the Xpert assay. Patients, the responsible medical team and study team will be masked to the study arm allocation (except for the study statistician, data managers and laboratory technicians). TB screening test results will be communicated to the medical teams responsible for clinical care of the patients (whilst maintaining masking to study arm allocation), but the clinical management decisions informed by the test results, including whether to commence TB treatment, will not be altered by the study team. Beyond collection of TB screening samples, running of assays and issuing of results, the study team will have no involvement in the clinical care of participants.

### Study population

The trial will take place at two sites: Zomba Central Hospital, Southern Malawi, and Edendale Hospital, KwaZulu-Natal, South Africa. Both hospitals serve populations with high HIV prevalence and TB incidence.

All new admissions to medical wards who have confirmed HIV-infection (either an existing or new diagnosis), irrespective of antiretroviral therapy (ART) status, will be screened for eligibility. HIV testing of all hospital admissions with unknown HIV-status is recommended as part of national guidelines at both trial sites, and will be supported by study staff where necessary. Eligibility criteria are designed to be as broad as possible. Patients will be eligible for inclusion regardless of reason for medical admission or presence of TB symptoms (unselected), although patients <18 years old, those who have received TB treatment in the preceding 12 months or isoniazid preventive therapy in the preceding 6 months, those unable to provide informed consent or living outside a pre-defined geographical area will be excluded. It is envisaged that recruitment will take place over a period of 24 months, with equal numbers recruited at each study site.

### Study procedures

After obtaining written informed consent from eligible patients, a study nurse will collect data at baseline to determine clinical history, including TB symptoms, past history of TB and HIV care, vital signs and nutritional and performance status. Venepuncture for haemoglobin concentration and CD4 cell count will be conducted and single sputum sample and up to 50mls of urine will be collected from all participants, irrespective of study arm. If sputum and/or urine samples cannot be spontaneously produced, participants will remain in the study but no further samples for TB investigation will be arranged by the study team. However, the responsible medical team will remain at liberty to arrange further TB investigations that are available as standard of care at the study hospital.

A randomisation list stratified by study site using computer generated random block size will be generated. Randomisation will occur at enrolment, but the study team will be masked to study arm allocation. When TB samples (sputum and/or urine) from participants arrive in the laboratory, the study laboratory technician will identify the allocated study arm by opening a sealed envelope marked with the unique participant ID, and determine which samples will be screened for TB. Urine from participants randomised to the standard of care arm will not be tested for TB and will be safely discarded. TB-LAM assays will be read by the laboratory technician and results will be deemed as positive using the grade 1 cut-off on the manufacturer’s reference card. 20 % of TB-LAM assays will read by a second, blinded reader for quality control.

TB screening tests will only be processed during office working hours on weekdays. Once the TB screening tests have been completed (anticipated to be within 24-48 h of sample collection), the results will be issued whilst maintaining masking of the study arm and responsible medical teams (reported as either ‘TB screening test positive’, ‘TB screening test negative’ or ‘TB screening test not done’). Rifampicin resistance results, when available, will also be communicated to the medical teams.

The responsible medical team will receive training on how to interpret the screening tests, including estimated positive and negative predictive values of the screening algorithms in each study arm, and that a negative screening test does not ‘rule out’ TB. The responsible medical team will be at liberty to organise further TB investigations and commence TB treatment as clinically appropriate. Management of HIV will be as per local and national guidelines, including timing of ART initiation. Data on TB investigations, diagnoses (including if microbiologically confirmed or ‘empirical’) and treatment will be collected during follow-up.

### Outcomes

The primary outcome is risk of all-cause mortality at 56-days following enrolment. Secondary outcomes are: time to all-cause mortality; proportions of patients with (i) microbiologically confirmed and (ii) clinically diagnosed TB disease; time to TB diagnosis and commencement of TB treatment; proportions of patients receiving antimicrobials and ART; and (i) duration of hospital admission, (ii) cumulative incidence of hospital readmission, and (iii) cumulative incidence of loss to follow-up. Outcomes will be ascertained by patient interview and review of medical notes during in-patient admission, and patient interview for vital status at 56-days post enrolment. This will be supplemented with information from patients’ next of kin for those lost to follow-up.

Microbiologically confirmed TB is defined as at least one positive smear-microscopy, Xpert, or culture positive result on any specimen or a positive urine TB-LAM result. Clinically diagnosed TB is defined as having a compatible clinical illness or radiological disease and/or the decision of the responsible clinical team to commence TB treatment in the absence of any positive microbiological tests for TB.

### Economic analysis

An economic analysis from a societal perspective will be undertaken to demonstrate the longer-term clinical and budgetary impact as well as the cost-effectiveness of the intervention; demonstrating economic feasibility and cost-effectiveness will be essential prior to implementation, should this screening intervention prove effective. Given the complexities of diagnosis and treatment of HIV/TB coinfection for patients requiring hospital admission, we will estimate health service costs based upon resource utilization of trial participants, inclusive of laboratory reagents and services, inpatient hospitalization days and outpatient visits, as well as drug costs. Trial-site specific costs will be collected on a limited cohort of 100 participants per site. We anticipate that, in the short term, costs of hospital admission are likely to account for a large proportion of health service provider expenditure; over the longer term, we expect increased survival related to the intervention will result in greater cumulative antiretroviral drug costs. Longer term cost-effectiveness will be estimated based on the Cost-Effectiveness of Preventing AIDS Complications-International (CEPAC-I) computer simulation model [29]. Model-based outcomes will include a short-term validation of trial results; that is, we will ensure that model results after 2 months of simulation accurately reflect trial outcomes of 56 days. Longer-term model results will include: 1-year and 5-year survival, TB-deaths averted, overall deaths averted and per person costs as well as life expectancy (LE) and lifetime costs. At each time horizon (1-year, 5-year and lifetime), the incremental cost-effectiveness of the intervention compared with the standard of care (Δ$/ΔLE, both discounted by 3 % per year) will be calculated. Model results will be examined in one-way and multi-way deterministic sensitivity analyses, examining influential parameters of interest (e.g. mean CD4 at presentation, TB screening test sensitivity/specificity and cost, active TB prevalence on admission). In addition to cost-effectiveness analyses, the budgetary impact of implementation and scale-up at 1- and 5- years of this intervention will be reported.

### Sample size justification

Sample size calculations were based upon unpublished data from the trial sites, which showed a mortality of HIV-positive medical in-patients of 21–23 % during hospital admission and are supported by subsequently published meta-analyses on hospitalised HIV-positive patients in Africa [2, 30]. We assumed that 56-day mortality would be 25–30 % in the standard of care arm and, based upon post-mortem prevalence of TB, we estimated 40–50 % of deaths in the standard of care arm would be TB-related.

The sensitivity of the intervention for diagnosing TB disease was assumed to be 80 % (a 3-fold increase in diagnostic yield compared to sputum alone) based upon a background study [14]. An observational study of early-initiation of TB treatment among HIV-positive smear-negative hospital in-patients observed a reduction in mortality at 2-months of 47 % in patients whose TB treatment was expedited by use of WHO-recommended diagnostic algorithm compared to standard practice [31]. Our study was powered to detect a 40–50 % reduction in TB-related mortality, and assuming half of deaths are TB related, this would equate to a 20–25 % reduction in all-cause mortality. Our sample size of 1300 patients per arm would provide 90 % power to detect a 25 % reduction in mortality and 80 % power to detect a 20 % reduction in mortality, assuming an all-cause mortality of 25 % in the ‘standard of care’ arm and a 2-sided type 1 error of 5 % and 10–15 % loss to follow-up. If the 56-day mortality was unexpectedly lower (20 %) in the standard of care arm, our sample size would still provide 80 % power to detect a 25 % reduction in all-cause mortality.

### Data collection and management

Data will be collected at four main times: enrolment, during the hospital admission, at hospital discharge and at the 56-day follow-up visit (Table 1). Specially designed case report forms are completed by study staff at each time point, and scanned, verified and committed to a local site database within 48 h of completion using the optical-character-recognition software TeleForm (Hewlett Packard Software, CA, USA). All data in critical fields are verified upon scanning. Completed forms are stored as the source documentation in a locked cabinet, with access restricted to specified study team members. Locator information is stored separately to other case report forms which are identified by unique participant ID number and do not contain any patient identifiable information. Queries based on data in the database are generated weekly, including date, range and logic checks, and sent to sites for resolution.

**Table 1:** Schedule of STAMP study activities

	Time-point:	Enrolment	Baseline assessment	TB testing	In-patient stay	Hospital exit	Outpatient Review
	Time On-Study:	Day 0	Day 0	Day 0–1	Day 0+	Day 0+	Day 56
Enrolment:							
Eligibility Screen		X					
Informed consent		X					
HIV confirmation		X					
Study arm allocation		X					
Interventions:							
Collect admission specimens (blood, sputum and urine)			X				
TB screening & issuing results				X			
Assessments:							
Baseline demographic and clinical information			X				
EQ5D			X				X
TB investigations, diagnosis and/or treatment					X	X	X
Vital status					X	X	X
HIV care/ART					X	X	X
Health service use					X	X	X

EQ5D quality of life questionnaire [38]

*ART* antiretroviral therapy

Follow-up at 56-days is undertaken through outpatient appointment attendance at the study site. If participants are unable to attend the outpatient appointment, they are traced by telephone and/or home visit if they have provided prior consent for this to occur. Participants are defined as lost to follow-up if they are unable to be contacted after three tracing attempts after 56-days from enrolment. Quality assurance processes are in place to check all consent forms, screening case report forms, laboratory case report forms and a random sub-sample of all other case report forms.

### Statistical analysis

**Fig. 2:**
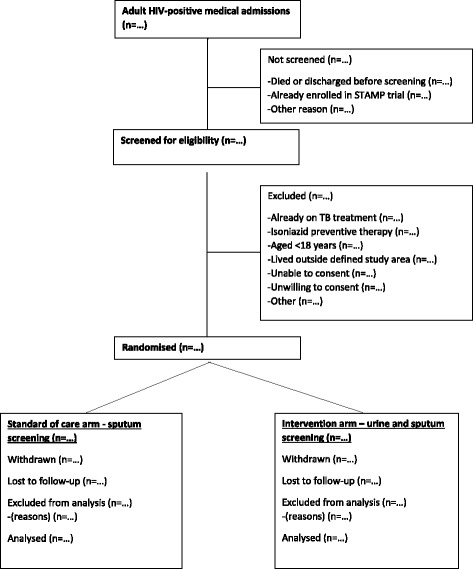
CONSORT flow diagram

The trial profile will be summarised using a CONSORT flow chart, including reasons for non-eligibility and non-enrolment (Fig. 2) [32]. All analyses will be conducted by initially assigned study arm in an intention-to-treat analysis, and adjusted for randomisation site. Baseline variables will be presented by study arm.

For the primary outcome the risk difference and odds ratio, and their associated 95 % confidence intervals for the effect of study arm on mortality risk will be calculated. The primary analysis of this endpoint will assume participants who are lost to follow-up before 56 days have not died. Sensitivity analyses will be conducted to assess assumptions made regarding missing data and patients lost to follow-up. A sub-group analysis of the primary outcome will be conducted for the following variables: study site, calendar time, baseline CD4 cell count (<100 or >100 cells/μL), presence of severe anaemia (Hb <80 g/L) and clinically suspected TB at admission. The interaction effects by subgroup will be investigated and p-values reported.

Secondary outcomes (as defined above) will be compared between study arms using logistic regression for binary outcome and survival analysis with Kaplan-Meier curves and Cox proportional hazards for time to event. All analyses will be adjusted for randomisation site.

A statistical analysis plan documents the analysis of all trial outcomes in detail, and was reviewed by the Data Safety and Monitoring Board (DSMB).

### Trial governance and approvals

The trial will be governed by a Trial Steering Committee (TSC), including an independent chairperson and at least three other independent members. The TSC will oversee the trial and advise the investigator team, including monitoring progress, receiving reports from the Data and Safety Monitoring Board (DSMB), and assessing the impact of new scientific evidence. The DSMB’s role is to protect the safety of trial participants, they will meet every six months to review data from the trial, as set out in the DSMB charter, and are independent to the trial sponsor (the London School of Hygiene & Tropical Medicine). Monitoring visits will be undertaken at least three-monthly for quality assurance and to ensure adherence to the trial protocol, and auditing of the trial conduct will be undertaken by the trial sponsor.

The trial has also received approval from the Research Ethics Committees of the London School of Hygiene & Tropical Medicine (ref: 9630), College of Medicine University of Malawi (ref: P.06/15/1743) and the University of KwaZulu-Natal Biomedical Research Ethics Committee (ref: BFC215/15). STAMP is registered with the ISRCTN Registry (ref: ISRCTN71603869) and the South African National Controlled Trials Registry (ref: DOH-27-1015-5185). The trial is funded by the Joint Global Health Trials scheme (a collaboration between the UK Department for International Development, the UK Medical Research Council and the Wellcome Trust), and was peer reviewed as part of the funding application process.

## Discussion

The STAMP trial aims to assess whether a novel, rapid urine-based screening strategy for TB with a high diagnostic yield can reduce early mortality in HIV-positive patients admitted to hospital in sub-Saharan Africa. The need for this trial is clear given the high mortality among HIV-positive patients admitted to hospital in these settings, and the high proportion of patients that die with undiagnosed TB disease [6, 30]. Positive urine-based TB diagnostic tests in advanced HIV are a marker of haematogenously disseminated renal TB, and these patients are at higher risk of mortality, supporting the rationale that earlier diagnosis and treatment may reduce mortality [33, 34].

Other trials have assessed empirical TB treatment in HIV-positive patients at high risk of TB, although mostly in outpatient settings. The REMEMBER trial failed to demonstrate a mortality benefit of empirical TB treatment compared to isoniazid preventive therapy [35]. The TB Fast Track trial assessed an algorithm using body mass index, presence of anaemia and urine TB-LAM testing to identify HIV-positive ambulatory patients with CD4 cell counts ≤150 cells/μL at high risk of TB in whom empirical TB treatment was started immediately, but found no reduction in 6-month mortality compared to the standard of care [34, 35]. The STAMP investigators believe that utilising new TB diagnostics can provide better specificity than empirical TB treatment and prevent the potential harm from prescription of TB treatment to HIV-positive patients without TB disease among hospital inpatients.

A trial of adjunctive urine testing with the TB-LAM assay in a selected population of hospitalised HIV-positive patients being investigated for TB (the LAM RCT) recently demonstrated a reduction in mortality at 2-months [26]. The STAMP trial differs from this RCT in respect to several factors. Firstly, the inclusion criteria differ in that STAMP is a screening intervention targeting unselected HIV-positive patients admitted to hospital regardless of clinical presentation, and the LAM RCT investigated the TB-LAM diagnostic in hospitalised HIV-positive patients who were presumed to have TB. As such, STAMP has the potential to detect TB among those without any clinical suspicion for TB on hospital entry. Secondly, the STAMP intervention includes the Xpert MTB/RIF assay performed on urine in addition to TB-LAM, whereas LAM RCT evaluated urine testing with TB-LAM alone. This increases the diagnostic yield of the intervention and adds the potential to diagnose rifampicin resistance. Thirdly, the standard of care in the STAMP trial includes Xpert testing of sputum (if produced) at all study sites, whilst the standard of care in LAM RCT varied from easy access to Xpert in South Africa to no access to Xpert in Zimbabwe. Finally, in the STAMP trial but not in the LAM RCT, both study teams and routine medical teams managing patients are masked to the study intervention arm (i.e. which TB screening tests are done).

The rationale for a clinical trial to demonstrate impact prior to implementation is supported by the lack of demonstrable benefit upon patient outcomes of sputum screening using Xpert in HIV-positive patients, despite increases in diagnostic yield compared to sputum smear microscopy [25]. Reasons for the failure of such trials of Xpert sputum testing to demonstrate impact may include lower occurrence of mortality endpoints in populations studies (largely outpatient based studies), high rates of empirical TB treatment and the failure of some positive Xpert results to be translated to TB treatment due to delays between submission of samples and issuing of results. The decision to start TB treatment is complex, and in such trials is likely to be influenced by the premise of the study to reduce undiagnosed, or ‘missed’ TB cases, potentially increasing empirical TB treatment [36].

These issues have been considered when designing the STAMP trial. For example, in-patient settings have a high mortality, and initiating TB treatment following positive results is more likely to occur when patients are admitted to hospital compared to community settings. Masking of clinicians, investigators and patients to the study intervention arm should reduce any ‘Hawthorne’ effect which could increase empirical TB treatment in the standard of care arm due to clinicians observing higher diagnostic yield in the intervention arm [37]. The use of ‘double-blinding’ in this manner is unusual in assessments of diagnostics, and, to our knowledge, this is the first time it has been used in TB diagnostics. However, a disadvantage of masking is that it might impair the ability of clinicians to make decisions about TB treatment in the absence of a positive screening result.

The STAMP trial sites have also been chosen to represent differing facilities, cadre of healthcare workers and resource utilisation in the region. Two studies to date, including one RCT, have demonstrated decreases in early mortality from expedited TB treatment in hospitalised HIV-positive patients, supporting the STAMP trial hypothesis that urine-based screening could reduce mortality [26, 31].

The unique challenges of ‘testing a test’ have also been considered in the STAMP study design, in particular, regarding the ethics of conducting such a trial. If considering the diagnostic accuracy or yield of the STAMP study intervention compared to sputum screening or clinical diagnosis alone, [14] it could be argued there is a lack of equipoise (i.e. disagreement about the relative merits of the intervention). Whilst equipoise may not exist for diagnostic accuracy of the intervention for detecting HIV-TB, equipoise clearly exists for the impact of the study intervention on early mortality in unselected HIV-positive hospital admissions when compared to the sputum and clinical screening strategies that are current standard of care [27]. We have also considered the potential negative consequences of a rapid microbiological TB diagnosis, such as delay in initiating ART, a reduction in the prescription of empirical antibiotics which may treat covert sepsis in this severely immunosuppressed population and false-positive rifampicin resistance results. This study will be able to describe and document the impact of these potential harms and compare these secondary endpoints between study arms.

Furthermore, the pragmatic nature of the STAMP trial, with clinical care including TB diagnostics outside the study intervention, TB treatment and ART under routine conditions for each study setting, make the findings more generalisable and applicable to scale up. The wide inclusion criteria and not restricting TB testing to patients with ‘presumptive’ TB (for which the definition often varies between settings) also makes the results more applicable. The recently published WHO guidance on the use of LAM lateral flow assays advises against its use for screening, based upon low quality evidence [22]. The STAMP study can provide randomised trial quality evidence to support or refute these recommendations for HIV-positive, hospitalised patients. The study intervention should be possible to replicate in resource-limiting settings given the widespread availability of the Xpert assay and lack of equipment required for the TB-LAM testing, in keeping with STAMP being a pragmatic trial. The concentration of urine prior to testing with the Xpert assay (which has been shown to increase sensitivity [14]) is done using a bucket centrifuge. Laboratory based research is also being undertaken to look for alternative, lower resource methods to concentrate urine prior to testing with Xpert.

The STAMP trial acknowledges the potential disadvantages of urine-based TB screening for this population, especially the cost implications. Although the intervention is relatively low-cost, health budgets in high TB and HIV settings are limited, and therefore widespread implementation of the STAMP study intervention may have substantial budgetary impact. The STAMP trial incorporates an economic analysis which will assess cost-effectiveness of the study intervention from a societal prospective and will include an analysis of budgetary impact of scale-up of this intervention. Should the intervention prove both effective and cost-effective, these analyses will assist in motivating the financial resources to promote its implementation.

In summary, the STAMP trial is assessing a novel, urine-based screening strategy utilising the TB-LAM and Xpert assays in HIV-positive patients admitted to hospital in sub-Saharan Africa. We hypothesise that use of this screening algorithm will reduce all-cause mortality at 2 months compared to sputum and clinical diagnostics (using the Xpert assay) which is the standard of care as currently recommended by the WHO. The trial commenced recruitment of patients in October 2015, and is projected to complete recruitment by September 2017 and follow-up by November 2017.

## Abbreviations

ART: Antiretroviral therapy
CEPAC-I: Cost-Effectiveness of Preventing AIDS Complications-International
DSMB: Data Safety and Monitoring Board
HIV: Human immunodeficiency virus
LAM: Lipoarabinomannan
LE: Life expectancy
RCT: Randomized Controlled Trial
TB: Tuberculosis
WHO: World Health Organization
