# Erratum to: Rapid urine-based screening for tuberculosis to reduce AIDS-related mortality in hospitalized patients in Africa (the STAMP trial): study protocol for a randomised controlled trial

**DOI:** 10.1186/s12879-016-1949-5

**Published:** 2016-10-25

**Authors:** Ankur Gupta-Wright, Katherine L. Fielding, Joep J. van Oosterhout, Douglas K. Wilson, Elizabeth L. Corbett, Clare Flach, Krishna P. Reddy, Rochelle P. Walensky, Jurgens A. Peters, Melanie Alufandika-Moyo, Stephen D. Lawn

**Affiliations:** 1 Department of Clinical Research, London School of Hygiene & Tropical Medicine, London, UK; 2 Malawi-Liverpool-Wellcome Trust Clinical Research Program, University of Malawi College of Medicine, Blantyre, Malawi; 3 Department of Infectious Disease Epidemiology, London School of Hygiene & Tropical Medicine, London, UK; 4 University of the Witwatersrand, Johannesburg, South Africa; 5 Dignitas International, Zomba, Malawi; 6 Department of Medicine, College of Medicine, University of Malawi, Blantyre, Malawi; 7 Department of Internal Medicine, Edendale Hospital, University of KwaZulu-Natal, Pietermaritzburg, South Africa; 8 Division of Pulmonary and Critical Care Medicine, Massachusetts General Hospital, Boston, MA USA; 9 Divisions of General Medicine and Infectious Disease, Massachusetts General Hospital, Boston, MA USA; 10 The Medical Practice Evaluation Center, Department of Medicine, Massachusetts General Hospital, Boston, MA USA; 11 Harvard University Center for AIDS Research, Harvard Medical School, Boston, MA USA; 12 The Desmond Tutu HIV Centre, Institute of Infectious Disease and Molecular Medicine, Faculty of Health Sciences, University of Cape Town, Cape Town, South Africa

## Erratum

The funding section in the original publication of this article [1] omitted the following information:

“KPR (grant number T32 AI007433) and RPW (grant number R37 AI093269) are funded by the National Institutes of Health (NIH). The report is solely the responsibility of the authors and does not necessarily represent the official views of the NIH.”
